# Photochemical Internalization with Fimaporfin: Enhanced Bleomycin Treatment for Head and Neck Cancer

**DOI:** 10.3390/pharmaceutics15082040

**Published:** 2023-07-28

**Authors:** Paula Enzian, Ramtin Rahmanzadeh

**Affiliations:** Institute of Biomedical Optics, University of Lübeck, 23562 Lübeck, Germany; p.enzian@uni-luebeck.de

**Keywords:** photochemical internalization, HNSCC, drug delivery, fimaporfin, bleomycin

## Abstract

Head and neck squamous cell carcinoma (HNSCC) still represents the world’s sixth most common tumor entity, with increasing incidence. The reachability of light makes HNSCC suitable for light-based therapies such as Photochemical Internalization (PCI). The drug Bleomycin is cytotoxic and used as an anti-tumor medication. Since Bleomycin is endocytosed as a relatively large molecule, part of it is degraded in lysosomes before reaching its intracellular target. The goal of our study was to improve the intracellular availability of Bleomycin with PCI. We investigate the intracellular delivery of Bleomycin after PCI with the photosensitizer Fimaporfin. A systematic variation of Bleomycin and Fimaporfin concentrations and light irradiation led to the pronounced cell death of HNSCC cells. After optimization, the same level of tumor cell death of 75% was reached with a 20-fold lower Bleomycin concentration. This would allow treatment of HNSCC with high local tumor cell death and reduce the side effects of Bleomycin, e.g., lung fibrosis, at the same time. This demonstrates the increased efficacy of the anti-tumor medication Bleomycin in combination with PCI.

## 1. Introduction

Head and neck squamous cell carcinoma (HNSCC) refers to a group of cancers that originate in the epithelium of the oral and nasal cavities, the pharynx, and the larynx [1]. HNSCC represents the world’s sixth most common tumor entity, with an incidence of about 500,000 new cases per year worldwide [2]. A total of 90% of head and neck cancers are squamous cell carcinomas. There has been an increase in the annual incidence of human papillomavirus (HPV)-related HNSCC, suggesting that a subset of HNSCC is a sexually transmitted disease with distinct clinical features. The prognosis, particularly in advanced tumor stages, is extremely poor. Unchanged during the past three decades, the overall 5-year survival rate is below 50% [3], since 50–60% of patients develop a recurrent disease [4]. Standard therapies are still surgery, radiotherapy, and chemotherapy.

For many therapeutic approaches, the delivery of the drug to the site of action is of fundamental importance for the drug’s effect. Many macromolecules, like antibodies, show low tissue penetration or cellular uptake and therefore low activity in vivo [5]. And moreover, not only the transport of active substances into cells but also their intracellular release from endosomes is limiting many therapeutic interventions [5,6].

A technique to improve the delivery of macromolecules is photochemical internalization (PCI), which involves the use of light and a photosensitizing agent [7,8]. Many macromolecules are taken up by cells via endosomes and will be degraded in lysosomes before reaching their intracellular target. Better delivery can enhance the effectiveness of drugs or other therapeutic agents. PCI is already in clinical trials for the release of gemcitabine in patients with bile duct cancer. In earlier studies, we showed the delivery of monoclonal antibodies into cells with the lipophilic photosensitizer Benzoporphyrin (BPD) [9,10,11]. More recently, we demonstrated the potential for successful delivery with photoactive liposomes [12].

In this study, we investigate the delivery of the cytotoxic drug Bleomycin into HNSCC cells after photochemical internalization with the photosensitizer Fimaporfin. Bleomycin is a water-soluble antibiotic with a size of approximately 1500 Da and belongs to the group of metallo-glycopeptide antibiotics discovered by Umezama et al. [13,14,15]. It is produced by the gram-positive bacterium *Streptomyces verticillus* [14,16,17]. For many decades, Bleomycin has been the subject of research and is clinically used as a chemotherapeutic agent against cancer. Bleomycin is used as a cancer chemotherapeutic agent to treat lymphoma, cervical and ovarian carcinoma, and other tumors. The biological effects of Bleomycin are thought to occur through metal-dependent oxidative cleavage of DNA and possibly RNA in the presence of molecular oxygen [16,18,19,20]. The resulting cytotoxicity is attributed to the double-strand and single-strand DNA breaks caused, which then lead to prolonged cell cycle arrest, apoptosis, and mitotic cell death [18,21]. Such double-strand breaks are important for anti-tumor activity [18,22].

However, a broad application of this active agent was prevented by the early development of drug resistance and dose-limiting pulmonary toxicity [16,18,20]. In consequence, there have been repeated attempts to develop new approaches in order to achieve better clinical efficacy with lower toxicity. Currently, Bleomycin is used in chemotherapy mostly in combination with other drugs to treat malignant germ cell tumors, Hodgkin’s lymphoma, and carcinomas of the skin, head, and neck [17,18,20,21].

Because it is a relatively large hydrophilic molecule, Bleomycin can only enter the cytosol to a limited extent and remains partially in the endosomes, which are subsequently degraded into lysosomes, so that Bleomycin cannot reach its intracellular target [23]. Due to these factors and the limitations of Bleomycin, PCI offers a promising approach. As a result, it often does not reach its intracellular target. Consequently, the Bleomycin molecule taken up by endocytosis requires release from endosomes in order to reach its specific intracellular target. This can be achieved by the simultaneous administration of an amphiphilic photosensitizer, which is incorporated into the plasma membrane and subsequently transferred to the inner endosomal membrane [24,25]. Upon light activation at appropriate wavelengths, the photosensitizer generates reactive oxygen species (ROS) that rupture the endosomal membrane, releasing the entrapped drug into the cytosol [24,26,27]. This delivery strategy is very promising for Bleomycin. Several studies are now investigating Bleomycin and various photosensitizers in combination to perform photochemical internalization [23,28,29,30,31,32,33].

We could show that photochemical internalization can strongly enhance the efficiency of Bleomycin in eliminating HNSCC cells. The use of PCI in the treatment of HNSCC is an interesting option to increase therapy efficiency and reduce the side effects of Bleomycin treatment at the same time.

## 2. Materials and Methods

### 2.1. Cell Culture

The UT-SCC-5 cell line used in this study is a squamous cell carcinoma (SCC) cell line from a tongue carcinoma. The cells were obtained directly from the University of Turku by Reida Grénmann. Cells were maintained in T-75 cell culture flasks (Greiner Bio-One, Kremsmünster, Austria) in Dulbecco’s Modified Eagle’s Medium (DMEM high glucose, Sigma-Aldrich, St. Louis, MO, USA). In addition, this culture medium was supplemented with 10% fetal calf serum (FCS, Sigma-Aldrich), 2% sodium pyruvate (100 mM, Sigma-Aldrich), 1% Antibiotic Antimycotic (with 10,000 units penicillin, 10 mg streptomycin, and 25 µg amphotericin B per mL, Sigma–Aldrich) and 1% MEM non-essential amino acids (Sigma-Aldrich). Cells were grown in a humidified incubator at 37 °C with a concentration of 5% CO_2_ and passaged at approximately 80% confluence.

### 2.2. Photosensitizer and Drug

Fimaporfin (TPCS_2a_) is a synthesized, amphiphilic photosensitizing molecule that can bind to the cell membrane [34]. Light-controlled activation of the photosensitizer leads to reactions with molecular oxygen (O_2_) and the formation of ROS species, primarily singlet oxygen [23,24,35]. The lifetime of singlet oxygen is short, and its range of action is small (10–20 nm) [23]. This enables the targeted destruction of the membrane of endosomes and lysosomes containing the photosensitizers using light while the contents of the organelles remain intact [36]. Subsequently, cytotoxic agents such as Bleomycin can be administered, which accumulate in the endosomal and lysosomal compartments. Reactive oxygen species can then damage the endo-/lysosomal membranes and release Bleomycin into the cytosol of the tumor cells.

**Figure 1:**
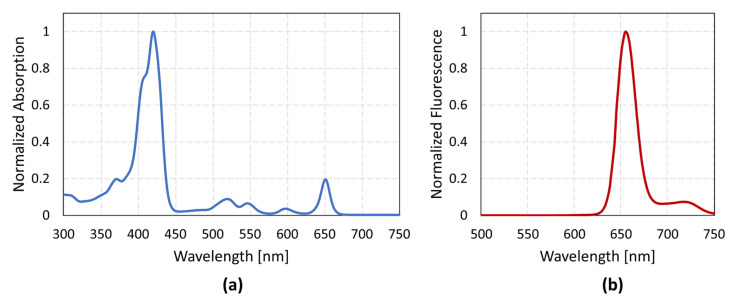
(**a**) Absorption spectrum of Fimaporfin. (**b**) Fluorescence spectrum at 420 nm excitation.

The absorption spectrum was measured with the spectrophotometer Hitachi U-2900 (Hitachi Ltd. Corporation, Tokyo, Japan). The measurement and normalization of the absorption spectrum of Fimaporfin revealed different peaks in the range of 350–700 nm (Figure 1a). The maximum absorption peak was detected at approx. 420 nm. However, in the longer wavelength range, a peak was also visible at 650 nm. A fluorescence spectrum was also recorded with SpectraMax M4 (Molecular Devices, San José, CA, USA) at an excitation wavelength of 420 nm. The fluorescence was between 640 and 680 nm (Figure 1b). In addition, an absorption spectrum of Bleomycin was recorded, which clearly shows only UV absorption and a peak at approx. 290 nm (Supplementary Figure S1). According to this, Bleomycin is not affected by PCI irradiation at longer wavelengths. The concentration of Bleomycin used in the subsequent experiments was based on the toxicity results from the UT-SCC-5 cells. For PCI irradiation experiments, concentrations of 0.1 µM and 0.25 µM were used, as these showed little to no self-cytotoxic effects in darkness and during irradiation without the presence of the photosensitizer.

### 2.3. Cell Viability Assay—MTT

UT-SCC-5 cells were seeded at a cell density of 4 × 10^4^ cells/mL in a 35 mm petri dish (Greiner Bio-One, Kremsmünster, Austria). 24 h after seeding, different concentrations of Fimaporfin were prepared in a medium and incubated on the cells for 18 h. For this purpose, the concentrations used (0.1 µg/mL; 0.155 µg/mL; 0.2 µg/mL; 0.4 µg/mL; 0.5 µg/mL) were prepared from a stock solution of 30 mg/mL TPCS_2a_ through dilution series. After the 18 h incubation period, the Fimaporfin-mixed medium was removed, and the cells were rinsed twice with Dulbecco’s phosphate-buffered saline (DPBS^+^ with MgCl_2_ and CaCl_2_, Sigma-Aldrich). A new culture medium was then added to the Petri dishes. Afterwards, cells were cultured in the incubator for an additional 48 h before being examined by MTT assay.

For this purpose, an MTT solution was prepared at a molar ratio of 1:1.5:1.5 consisting of MTT Stock solution (4 mg/mL): DPBS^+^: medium. This solution was incubated with 500 µL in each dish for 2 h. The MTT solution was then removed, and an additional incubation of 20 min with dimethylsulfoxid (DMSO, Sigma-Aldrich) on a shaker was performed to dissolve the formazan crystals. Three technical replicates of 50 µL were collected from each petri dish and transferred to a 96-well plate (Greiner Bio-One). Absorbance was measured using a microplate reader (SpectraMax M5, Molecular Devices) at 570 nm. The absorbance of the treated samples compared to the absorbance of the untreated controls was used as a measurement of cell viability. The viability data of the different groups were expressed as the mean and standard deviation of a total of three measurements with three petri dishes each.

### 2.4. Cell Survival Assay—CFA

In order to investigate the survival and proliferation ability of UT-SCC-5 after treatment with the chemotherapeutic agent Bleomycin, a colony formation assay (CFA) adapted from Franken et al. [37] was performed. For this purpose, the method of “plating before treatment” was used to investigate the sensitivity of cells to different treatment options. Initially, UT-SCC-5 cells were harvested from a stock culture and seeded at the appropriate dilution with the desired seeding concentration (between 100 and 500 cells per well, depending on the sample) in 6-well plates (Sarstedt, Nümbrecht, Germany) or 35 mm petri dishes (Greiner Bio-One). The respective treatment was carried out 24 h after seeding. After treatment, the dishes were placed in an incubator and incubated for 12 days so that at least six potential cell divisions could occur.

Treatment options are divided into three groups (A, B, and C), each with two subgroups (Table 1). Treatment A was used to test the toxicity of Fimaporfin. For this purpose, 24 h after seeding the cells, TPCS_2a_ was incubated for 18 h at different concentrations. Subsequently, the samples were washed twice with DPBS^+^ and supplied with fresh culture medium. According to the subgroups, either cells were cultured again in the incubator without irradiation (A1) for 12 days or they were irradiated at this time point (A2). The irradiation parameters can be seen in Table 1. For all irradiations at a wavelength of 650 nm, the ML660 laser system (Modulight, Tampere, Finland) was used.

Treatment B was also distinguished between “without irradiation” (B1) and “with irradiation” (B2). In these experiments, 24 h after seeding, Bleomycin was incubated at various concentrations for 4 h. Subsequently, these samples were also rinsed twice with DPBS^+^ and then supplied with a new medium.

In the third treatment method (C), treatments A and B were combined. 24 h after seeding, UT-SCC-5 was incubated with Fimaporfin for 18 h, followed by rinsing twice with DPBS^+^ and incubation with Bleomycin for 4 h. Afterwards, the samples were again rinsed twice with DPBS^+^ and supplied with a new medium. A distinction was also made between irradiated samples (C1) and non-irradiated samples (C2).

After 12 days, the colonies were fixed and stained in all samples. The fixation was carried out on the ice. The medium was removed, the samples were rinsed with cold DPBS^+^, and each well was incubated with 2 mL of ice-cold methanol for 10 min. After the methanol was removed, the plates or petri dishes were taken off the ice and allowed to come to room temperature for 15 min. Colonies were incubated with 0.5% Crystal Violet for 10–15 min on the orbital shaker. Then the staining solution was removed, and the plates were carefully rinsed with clear water. This was left to dry overnight at room temperature and subsequently counted.

A stereomicroscope (WILD Heerbrugg, WILD M8, Heerbrugg, Switzerland) was used to count colonies. A cell cluster is counted as a colony if it consists of at least 50 cells. The plating efficiency (PE) of the untreated control cells was then determined:

(1) $$\mathrm{PE}=\frac{\mathrm{no}.\;\mathrm{of}\;\mathrm{colonies}\;\mathrm{formed}}{\mathrm{no}.\;\mathrm{of}\;\mathrm{cells}\;\mathrm{seeded}\;}\times \;100\%$$

PE was independently re-determined in each experiment since small variations in conditions can affect this factor. The surviving fraction (SF) of cells after a treatment is always calculated under consideration of the PE values obtained from the control cells.

(2) $$\mathrm{SF}=\frac{\mathrm{no}.\;\mathrm{of}\;\mathrm{colonies}\;\mathrm{formed}\;\mathrm{after}\;\mathrm{treatment}}{\mathrm{no}.\;\mathrm{of}\;\mathrm{cells}\;\mathrm{seeded}\;\times \;\mathrm{PE}}$$

### 2.5. TuBB-9 Antibody and FITC Labeling

To examine the intracellular delivery proof-of-concept by fluorescence microscopy, the monoclonal antibody TuBB-9 was labeled with the fluorescent dye fluorescein isothiocyanate (FITC). The antibody is diluted 1:5 in a sodium carbonate buffer consisting of 160 nM Na_2_CO_3_ and 333 nM NaHCO_3_ with pH 9.3 to obtain an antibody concentration of 1 mg/mL. The suspension is centrifuged for 20 min at 1855× *g,* and the residues remaining in the filter are taken into buffer solution (pH 9.3). Subsequently, 1 mg/mL of FITC (dissolved in DMSO) is added to the TuBB-9 solution. The resulting suspension is incubated at room temperature with constant agitation for 2 h to obtain FITC binding to TuBB-9.

Using a Sephadex column (NAP-25, GE Healthcare Life Sciences, Chicago, IL, USA), which has been buffered with tris-buffered saline (TBS) (pH 8.2), the obtained sample is purified. The eluate is again centrifuged. The sample was removed from the filter, and to wash out any remaining residue in the filter, it was rinsed twice with 500 µL TBS (pH 7.5), and then addition was carried out to the sample. Finally, the concentration of the antibody and the fluorescent dye is determined using an absorption spectrum.

### 2.6. Cellular Uptake and Endosomal Release of the Antibody

Cellular uptake and localization of TuBB-9-FITC and Fimaporfin were observed in UT-SCC-5 cells by fluorescence microcopy (TE-Eclipse, Nikon, Tokyo, Japan). For this purpose, the cells were seeded with a cell density of 60,000 cells/mL in glass bottom petri dishes (Greiner Bio-One). 24 h after seeding, the cells were incubated in DMEM containing 0.1 µg/mL Fimaporfin for 18 h. Cells were washed twice with DPBS^+^ and incubated for four hours with the TuBB-9-FITC construct. The cells were washed again twice with DPBS and then supplied with the new medium. Irradiation for ROS production and endosome disruption took place at 420 nm (LED, Roithner, Wien, Germany) and 0.25 J/cm^2^. Fluorescence microscopy was performed before irradiation and 30 min or 24 h after irradiation to observe the release from endosomes and their localization of TuBB-9-FITC conjugates.

## 3. Results

### 3.1. TPCS_2a_ without Light Irradiation

**Figure 2:**
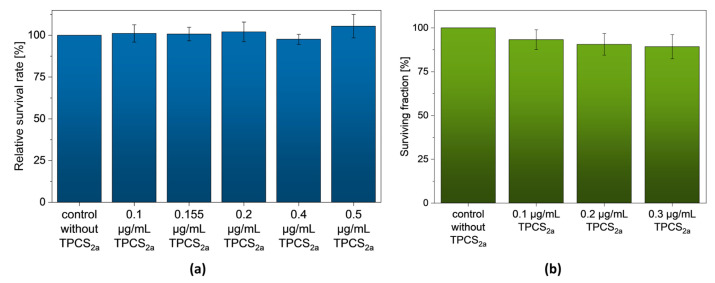
Fimaporfin without light irradiation shows no cytotoxicity against UT-SCC-5 cells. (**a**) MTT assay: 48 h after incubation of different Fimaporfin concentrations (0.1–0.5 µg/mL) without irradiation. (**b**) CFA: 12 days after incubation of different Fimaporfin concentrations (0.1–0.3 µg/mL) without light irradiation.

In order to verify the dark toxicity of the photosensitizer Fimaporfin (TPCS_2a_), it was first incubated without light irradiation on UT-SCC-5 cells. The MTT assay showed a high survival rate of the cells 48 h after incubation with different Fimaporfin concentrations (0.1–0.5 µg/mL). Furthermore, long-term toxicity could be excluded by the colony-forming assay (CFA) 12 days after incubation with Fimaporfin (Figure 2b). The surviving fraction at a concentration of 0.3 µg/mL was still 89.26 ± 6.89%, and with a concentration of 0.2 µg/mL, it was possible to achieve a surviving fraction of 90.59 ± 6.15%. It can be assumed that the UT-SCC-5 maintain their reproductive integrity for a longer period after the addition of TPCS_2a_. Therefore, Fimaporfin is not toxic to UT-SCC-5 cells without irradiation up to a concentration of 0.3 µg/mL.

### 3.2. Bleomycin without Light Irradiation

**Figure 3:**
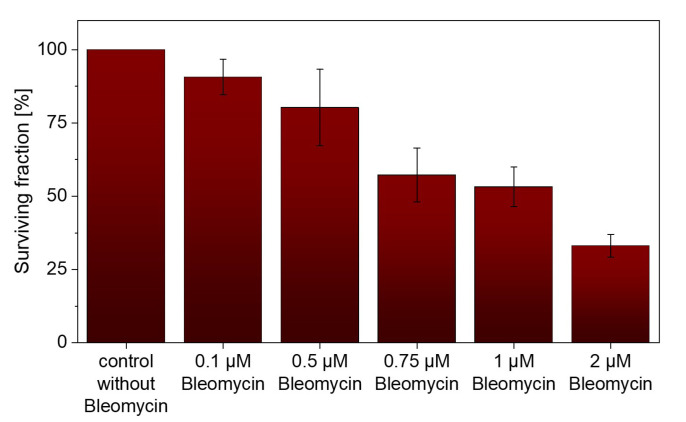
12 days after incubation of Bleomycin the CFA assay showed high surviving fraction of UT-SCC-5 cells up to a concentration of 0.5 µM.

The chemotherapeutic agent Bleomycin was also tested at different concentrations without light irradiation for its cytotoxic effect on UT-SCC-5. Compared to the untreated control group, a high surviving fraction of 80.27 ± 12.99% was observed 12 days after incubation with Bleomycin up to a concentration of 0.5 µM (Figure 3). With a concentration of 2 µM, the surviving fraction decreased to 33.12 ± 3.86%.

### 3.3. TPCS_2a_ in Combination with Light Irradiation

After proving that both substances did not induce cell-damaging effects at low concentrations under dark conditions, toxicity under light irradiation was investigated. For this purpose, a CFA was performed after 12 days for both Fimaporfin and Bleomycin.

**Figure 4:**
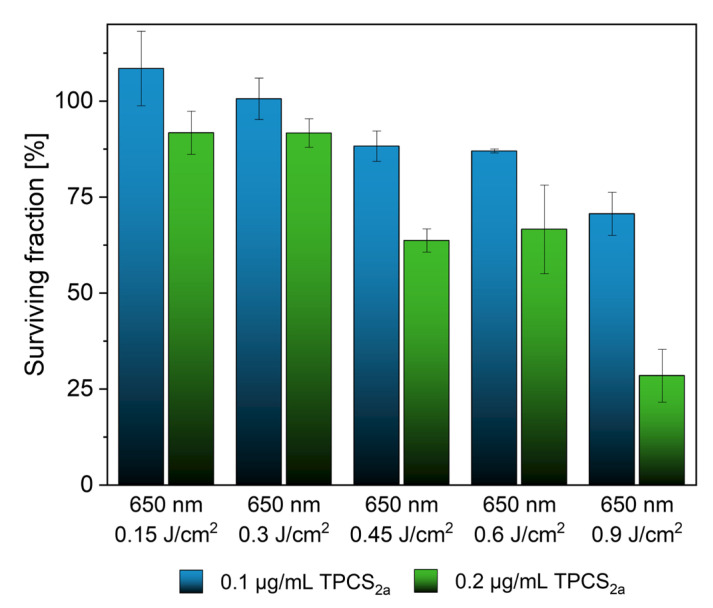
CFA assay showed a survival rate of 93.14 ± 2.93% at a concentration of 0.2 µg/mL TPCS_2a_ and an irradiation energy of 0.3 J/cm^2^. At higher irradiation energies, the survival rate decreased continuously.

Fimaporfin (0.1 and 0.2 µg/mL) was incubated with UT-SCC-5 cells for 18 h and then irradiated at 650 nm at different energies. At a concentration of 0.1 µg/mL TPCS_2a_, a high survival rate of 87.98 ± 1.68% was observed with up to 0.6 J/cm^2^ (Figure 4). At a twofold higher Fimaporfin concentration, a surviving fraction up to 93.14 ± 2.93% could still be observed at 0.3 J/cm^2^. However, at a concentration of 0.2 µg/mL, the survival rate continuously decreased at higher exposure energies. Consequently, we demonstrated the toxic limits of Fimaporfin under irradiation for UT-SCC-5 cells.

### 3.4. Bleomycin with Irradiation

**Figure 5:**
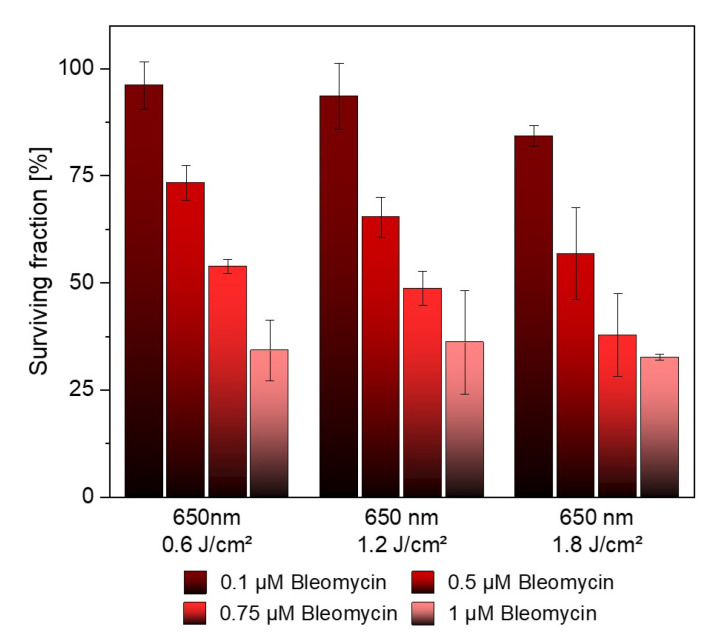
12 days after irradiation, CFA assay showed a high surviving fraction of UT-SCC-5 cells at a Bleomycin concentration of 0.1 µM at all three energies.

Furthermore, the cytotoxic effect of Bleomycin on UT-SCC-5 cells under irradiation was also investigated. Different concentrations up to 1 µM were analyzed after 12 days by CFA. At a concentration of 0.1 µM, high survival rates (96.11–84.28%) were observed at all three irradiation energies (0.6–1.8 J/cm^2^). However, higher concentrations of Bleomycin showed significant toxicity (Figure 5).

### 3.5. Combination of TPCS_2a_ and Bleomycin with Light Irradiation

To test the potential of photochemical internalization of Bleomycin on tumor cell toxicity. Bleomycin and Fimaporfin were incubated with UT-SCC-5 cells and irradiated with the previously determined irradiation parameters. Fimaporfin was applied at a concentration of 0.2 µg/mL throughout the experiments. 

**Figure 6:**
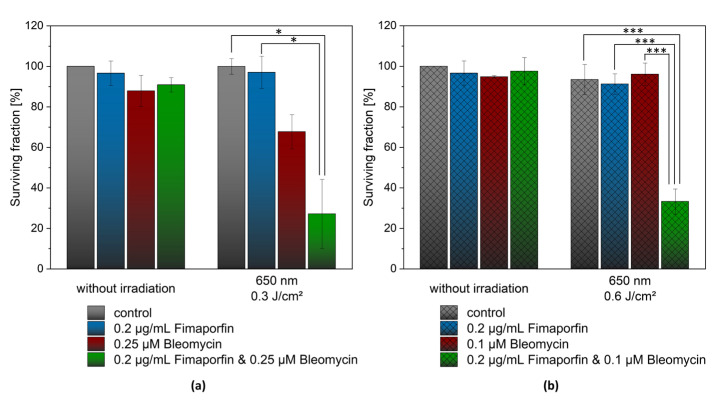
CFA 12 days after irradiation with the combination of Fimaporfin and Bleomycin. (**a**) Setup using low irradiation energy of 0.3 J/cm^2^ and Bleomycin in a concentration of 0.25 µM. (**b**) Setup with lower concentration of Bleomycin (0.1 µM) but an irradiation energy of 0.6 J/cm^2^. The two linked columns from the *t*-test analysis indicate the statistically significant difference (* *p* ≤ 0.05 significant; *** *p* ≤ 0.001 highly significant) in cell viability between the two samples.

Figure 6 shows the significant and selective cytotoxicity of the combination treatment. Two different Bleomycin concentrations and light energy setups were investigated. Using a low irradiation energy of 0.3 J/cm^2^ and Bleomycin in a concentration of 0.25 µM, the combined treatment resulted in a surviving fraction of 27.1 ± 17.1% after 12 days (Figure 6a). However, the use of Bleomycin alone with irradiation showed a reduced survival rate of 67.7 ± 8.5%. In contrast, TPCS_2a_ (0.2 µg/mL) alone still showed a high survival rate at an irradiation energy of 0.3 J/cm^2^.

In the second setup (Figure 6b), at a lower concentration of Bleomycin (0.1 µM) but an irradiation energy of 0.6 J/cm^2^, the surviving fraction was 96.11 ± 5.54% without light application. The combination of both components and irradiation led to a significant decrease in the surviving fraction to 33.22 ± 6.22%. Interestingly, in this setup, when Fimaporfin was administered alone and irradiated, more than 90% of cells survived.

High significance (*p* ≤ 0.001) was demonstrated between Bleomycin irradiated alone and the combination of both agents with irradiation. Furthermore, a significant difference in the viability of the cells was also found between irradiation with Fimaporfin alone and the combination of both agents. A synergistic effect can be demonstrated for the cytotoxic effects of the two agents in combination.

### 3.6. Microscopy of TPCS_2a_ and TuBB-9-FITC after Irradiation

**Figure 7:**
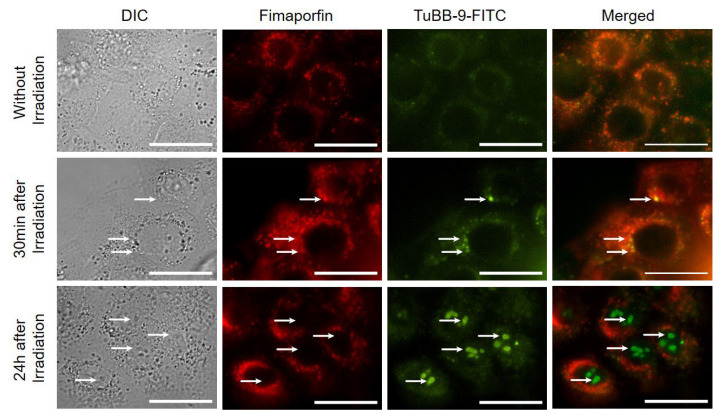
The monoclonal antibody TuBB-9-FITC can be delivered intracellularly with photochemical internalization using Fimaporfin. Microscopy images of TPCS_2a_ and TuBB-9-FITC without and after irradiation. Irradiation took place at 420 nm and 0.25 J/cm^2^. Fimaporfin was used at a concentration of 0.1 µg/mL. Before irradiation, the antibody was mainly located in spots in the endosome (**upper row**). 30 min after irradiation, the antibody was visible next to the nucleoli (arrows, **middle row**). 24 h after irradiation, it can be detected in the nuclei (arrows, **lower row**). Scale bar: 25 µm.

We further demonstrated that not only Bleomycin but also monoclonal antibodies can be delivered intracellularly with photochemical internalization using Fimaporfin. For this study, UT-SCC-5 cells were incubated first with Fimaporfin for 18 h and then for 4 h with the antibody TuBB-9-FITC against the nuclear protein Ki-67. Before irradiation, the antibody was mainly located in spots in the endosome (Figure 7, upper row). 24 h after light irradiation, the antibody was located at the site of its target protein, Ki-67, inside the nucleoli (Figure 7, lower row). Further studies need to follow to improve the therapeutic approach against HNSCC in the future.

## 4. Discussion

In this study, we showed the applicability of photochemical internalization with the clinically relevant drugs Fimaporfin and Bleomycin in the HNSCC cell line UT-SCC-5. Moreover, we demonstrated the use of this approach for the intracellular delivery of monoclonal antibodies. Fimaporfin alone showed no cytotoxicity without light at the concentrations used (up to 0.5 µg/mL). Other studies have also shown that Fimaporfin had little cytotoxic effect on other cell lines without irradiation [38]. However, toxic effects on the cell can occur even when the photosensitizer is administered alone when combined with irradiation. The higher either the irradiation dose or the Fimaporfin concentration, the stronger the toxicity towards the UT-SCC-5 cells becomes already at the first irradiation.

If Bleomycin is administered alone, a high concentration of 2 µM is needed to provoke 75% cell death. In other cancer cell lines, comparable behavior has been demonstrated, requiring high concentrations of Bleomycin alone to induce efficient cell death [39,40]. Since the primary mechanism of Bleomycin is to oxidatively damage DNA by binding to metal ions and forming metallo-Bleomycin complexes, it produces the reactive oxygen species that cause DNA single and double strand breaks [41]. This inhibits the progression of cell replication and, thus, the growth of cancer cells [18]. For this reason, when choosing the dosage of Bleomycin, it is necessary to control the concentration at which damage to the cells occurs.

A systematic investigation of different Fimaporfin and Bleomycin concentrations in combination with different irradiation energies resulted in optimal treatment conditions showing 75% cell death with only 0.1 µM Bleomycin. This same cell elimination rate with a 20-fold decreased Bleomycin concentration is most likely made possible due to improved Bleomycin delivery inside the cell. Bleomycin, as a relatively large molecule, stays in endosomal vesicles after cellular uptake and will be degraded in lysosomes without reaching its intracellular target (e.g., DNA) [23,28]. Consequently, the effect of Bleomycin is severely restricted [28,42]. Results of the study demonstrated that using Bleomycin in combination with PCI significantly increased cytotoxicity in vitro compared to Bleomycin on its own. Due to the light-regulated activation of Fimaporfin and its associated formation of ROS species, the membrane of the endosomes or lysosomes is specifically destroyed due to the small action radius of singlet oxygen. Through this targeted opening, a synergistic effect on the intracellular availability of Bleomycin can be achieved, followed by increased cancer cell death.

In addition, we demonstrated that the effects of PCI treatment were stronger in the UT-SCC-5 cells in comparison to a control group of fibroblasts (Supplementary Figure S2). Using the MTT assay to detect early toxicities, the combination of Fimaporfin and Bleomycin together with irradiation at 650 nm was shown to reduce the survival rate by up to 36% in UT-SCC-5 cells compared to the single administration of Bleomycin. In contrast, only a 20.6% reduction in cell viability was detected in fibroblasts. This resistance to Bleomycin in normal tissues may indicate the presence of the enzyme bleomycin hydrolase, which belongs to the cysteine proteinase family [43,44]. This enzyme may inhibit cytotoxic activity by reducing iron binding and preventing the cellular effects mentioned above [43]. There are studies on the ability of the expression of this enzyme to prevent Bleomycin-induced cellular damage [14,43,44,45,46,47]. However, no definitive conclusions on this relationship have been reached to date.

Reducing the dose of Bleomycin with the same local effect on tumor cells is very interesting with regard to the severe side effects of Bleomycin treatment. One of the main problems associated with the use of Bleomycin is pneumonitis, an inflammation of the lung, which affects more than 10% of patients [48,49,50]. This can lead to fibrosis and breathing problems. Also, other side effects like inflammation and thickening of the skin, fever, hair loss, or mucositis could be reduced [50].

In addition, it could be shown that it is also possible to deliver antibodies specifically to the target site in head and neck cancer in combination with Fimaporfin. The low concentration of 0.1 µg/mL has minimal cytotoxic effects on the cells. Fimaporfin should also be further investigated as a possible therapeutic tool for antibody delivery to the nuclei.

## 5. Conclusions

Our study shows the potential of Photochemical Internalization with Fimaporfin for the delivery of Bleomycin in HNSCC cells, leading to tumor cell death. With PCI, the dose of Bleomycin was 20-fold lower to provoke the same amount of cell death as without PCI. The findings of this study support the idea that PCI may be a valuable tool for the treatment of head and neck cancer.

## Figures and Tables

**Table 1:** Irradiation parameters for the different treatment groups and the used concentrations of Fimaporfin and Bleomycin.

Treatment Group		Irradiation Parameters		Fimaporfin Concentration [µg/mL]	Bleomycin Concentration [µM]
		Wavelength [nm]	Exposure Energy [J/cm^2^]		
Fimaporfin	A1	no Irradiation	-	0.1/0.155/0.2/0.3/ 0.4/0.5	-
	A2	650 nm	0.15/0.3/0.45/0.6/0.9	0.1/0.2	-
Bleomycin	B1	no Irradiation	-	-	0.1/0.5/0.75/1/2
	B2	650 nm	0.6/1.2/1.8	-	0.1/0.5/0.75/1
Combination	C1	no Irradiation	-	0.2	0.1/0.25
	C2	650 nm	0.3/0.6	0.2	0.1/0.25

## Data Availability

Data will be made available on request.

## Supplementary Materials

The following supporting information can be downloaded at: https://www.mdpi.com/article/10.3390/pharmaceutics15082040/s1. Figure S1: Normalized absorption spectrum of Bleomycin. Figure S2: (a) MTT assay of UT-SCC-5 cells: 48 h after irradiation with 650 nm and an irradiation energy of 0.6 J/cm². (b) MTT assay of fibroblasts: 48 h after irradiation with 650 nm and an irradiation energy of 0.6 J/cm².
