# Supplementary material for: Photochemical Internalization with Fimaporfin: Enhanced Bleomycin Treatment for Head and Neck Cancer
Source: Pharmaceutics. 2023 Jul 28;15(8):2040. doi: 10.3390/pharmaceutics15082040 (PMC10458762; doi:10.3390/pharmaceutics15082040)
Supplement: Supplementary file 1 [file pharmaceutics-15-02040-s001.zip › pharmaceutics-2443748-supplementary.pdf]

<u>Supplementary material</u>

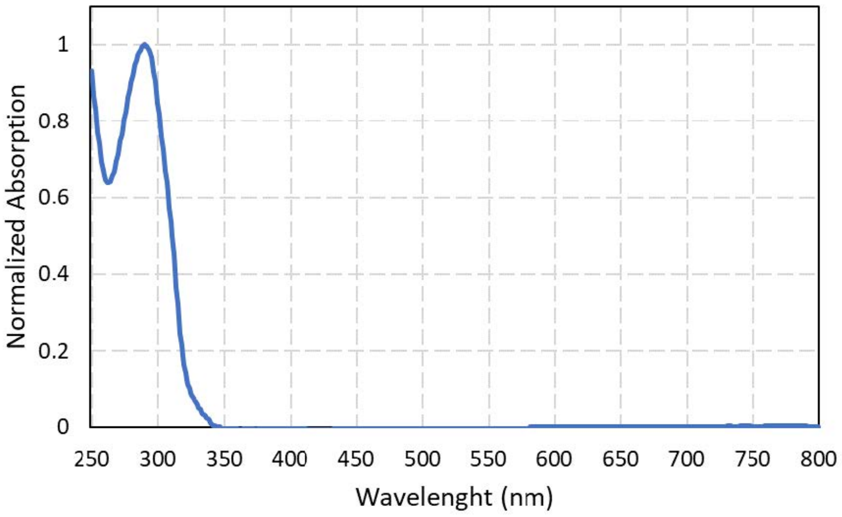

Supplementary Figure S1. Normalized absorption spectrum of Bleomycin.

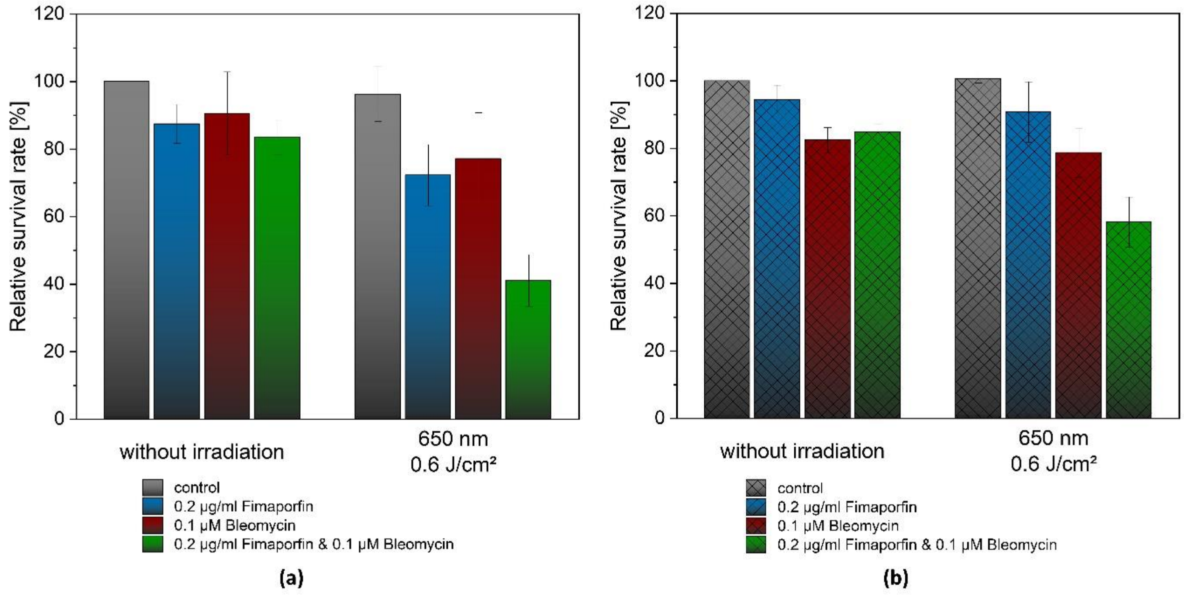

Supplementary Figure S2. (a) MTT assay of UT-SCC-5 cells: 48 h after irradiation with 650 nm and an irradiation energy of 0.6 J/cm². **(b)** MTT assay of fibroblasts: 48 h after irradiation with 650 nm and an irradiation energy of 0.6 J/cm².
